# Discussing physical restrictions in care plan meetings between family members of residents with dementia and nursing home staff

**DOI:** 10.1177/14713012231186346

**Published:** 2023-06-30

**Authors:** Jenny Paananen, Camilla Lindholm

**Affiliations:** Department of Nursing Science, 8058 University of Turku, Finland; Faculty of Information Technology and Communication Sciences, 7840 Tampere University, Finland

**Keywords:** dementia, decision-making, restraining, interaction, family members, nursing home, care plan, care plan meeting

## Abstract

In long-term dementia care, caregivers face a dilemma. On the one hand, they need to respect the residents’ right to self-determination, but on the other hand, they sometimes rely on physical restraints to deal with potential violence and self-destructive behavior. The issue of self determination is further complicated by residents often depending on family members as advocates in decision-making. In this article, we examine 15 care plan meetings to identify the professional practices of discussing the physical restrictions posed to residents with severe dementia. Our method is conversation analysis. Our analysis demonstrates that staff members’ practices involve informing, accounting, and agreeing on the goals rather than on the methods of physical restraining. Staff members tend to first inform family members about the principles of restraining and then account for the use of restraints. Accounts highlight the problems that can be avoided and the benefits that can be achieved by limiting residents’ actions. Consequently, the family members’ role in the discussion is to accept the decision that has already been approved by authorities. As the staff members highlight the aim of protecting the well-being of the resident, the family members tend to respond with overt agreement and even promote the use of restraints. Current negotiation practices provide insufficient opportunities for family members to advocate for residents. Therefore, we recommend involving family members in decision-making about restraining at an earlier stage, adjusting the protocol in care plan meetings, and engaging the family in minimizing and preventing restraints. In general, staff members should pay more attention to the residents’ experiences and the family members’ lifeworld knowledge of the residents.

## Introduction

The self-determination of a person is a core value in healthcare: patients should be respected as individuals, and their values, experiences, and wishes should be considered in decisions about their care and treatment. Dementia care in nursing homes is no exception, but advanced dementia can affect a person’s ability to communicate their views. In late-stage dementia, language use and gestural communication are typically limited, and some individuals are only able to produce nonverbal vocalizations or communicate with the gaze (Hydén, 2011; Kim & Bayles, 2007). Dementia can also cause difficult behavioral symptoms that may affect an individual’s self-determination. For example, in a nursing home environment, violent and self-destructive actions may lead to the use of physical and chemical restraints and the omission of utilities. Physical restraints include, for example, safety belts, bedside rails, bodysuits, vests, and locked doors (see Gastmans & Milisen, 2006).

There is vast literature on the negative consequences of physical restraints in dementia care (for an overview, see De Bellis et al., 2013). Limiting residents’ mobility is associated with poor health outcomes, such as functional decline, cardiovascular stress, and muscle atrophy, increasing the risk of serious physical injuries and death. Complications for residents also include psychological and emotional consequences, such as feelings of shame, agitation, anxiety, and social isolation (De Bellis et al., 2013; Gastmans & Milisen, 2006). Family members have conflicted feelings of restraint, considering it a ‘necessary evil’ for the resident’s safety (De Bellis et al., 2013, p. 99). The practice of restraining poses serious ethical challenges for nursing staff. Professional caregivers often experience a dilemma between the use of restraints and the violation of residents’ autonomy (Chuang & Huang, 2007). However, despite these negative feelings, caregivers often decide to use restraints in daily practice (Möhler & Meyer, 2014). The literature tends to provide low staffing levels as an explanation for the use of physical restraints. However, these explanations were not supported in the reports examined by De Bellis et al. (2013) Instead, they indicated that inaccurate knowledge was a primary factor in the use of restraints. In fact, restraints consume resources, as restrained residents require more nursing care and their care costs more than the care of other residents in similar conditions (Philips et al., 1993). Given the serious outcomes of restraints, a general consensus is that their use should always be minimal and that the choice to use them should be based on individual assessments (see, e.g., Gastmans & Milisen, 2006).

This paper focuses on how staff members in Finnish nursing homes inform residents’ family members about and account for the need for physical restraints, and how family members, who act as the residents’ advocates, are involved in decision-making about restraints. In Finnish long-term care, the use of restraints requires a doctor’s written license. Restraining must be justified as necessary for the health or safety of the resident, and the license is always temporary. The use of restraints must also be documented (Act on Supporting the Functional Capacity of the Older Population and on Social and Health Care Services for Older Persons 980/2012; Valvira, 2021). However, there is no detailed legislation on the principles of restraining; therefore, the methods and guidelines vary between facilities in Finland.

From the viewpoint of the rights of residents with dementia, family members’ involvement in decision-making about care is not only meaningful but also implied by Finnish law: “If a patient because of mental disorder or mental disability or for other reason cannot decide on the treatment given to them, the legal representative or a family member or other close person of the patient has to be heard before making an important decision concerning treatment to assess what kind of treatment would be in accordance with the patient’s will.” (Act on the Status and Rights of Patients, 785/1992, p. § 6). Thus, if the use of restraints is considered an important decision concerning the treatment of a nursing home resident living with late-stage dementia, restraining should be negotiated with a family member or other representative—and in terms of the resident’s wishes and preferences. This paper, however, demonstrates that, interactionally, the current practice does not involve such negotiation but consists of informing, accounting, and agreeing on the goals of restraining rather than the methods used to achieve them.

## Decision-making involving individuals living with Dementia

Recent decades have demonstrated an increasing body of research on decision-making in health care, focusing on aspects such as patient self-determination and patients making decisions in collaboration with medical professionals. Joint decision-making relates to the ideal of patient-centered care (Daly et al., 2018), which is a well-established philosophy in dementia care (Kitwood, 1997). However, models for joint decision-making usually stress the participants’ capacity to make rational decisions (Peisah et al., 2013). Therefore, these models are not necessarily suitable in situations of decision-making involving one or several participants with cognitive or interactional challenges. Previous research has demonstrated that the participation of persons living with dementia in joint decision-making varies (Mariani et al., 2017) and decreases as the disease progresses (Miller et al., 2016). As dementia progresses, family members are frequently called upon to make choices about treatment and care (Caron et al., 2005). However, individuals with dementia may also be prematurely excluded from making decisions about their care (Miller et al., 2016). Individuals with dementia tend to participate more frequently in small-scale decisions, such as daily activities, than in large-scale decisions, such as medical care (Smebye et al., 2012). Occasionally, they delegate decision-making to their family members and are satisfied with the arrangement (Smebye et al., 2012). As demonstrated in previous research, family members play a primary role in clinical decision-making to ensure the best care for their loved ones, reporting the experience of acting as advocates for residents’ needs (Fetherstonhaugh et al., 2021).

## Accounts in interaction

In our data, nursing staff members typically use accounts alongside informing when discussing restraining with residents’ family members. *Accounts* are actions that propose a cause and seek to present something as legitimate or ordinary (Sacks, 1992, lecture 1; Scott & Lyman, 1968; Antaki, 1994). In a sense, accounts deal with explicit or underlying *Why?* questions in interaction. They explain the speaker’s motives and signal acknowledged responsibility, that is, accountability. The function of accounts is to foster agreement between interlocutors or repair fractured social interactions. Accounts are often given for dispreferred actions (Firth, 1995; Heritage, 1990), such as being late from a meeting (*the bus got stuck in the traffic*) or refusing an invitation or a request (*I’m afraid I’m working on Saturday*) (see also Schegloff, 2007, p. 65).

However, accounts do not necessarily bear associations with the speaker’s troublesome conduct. In institutional encounters, accounts are often used to minimize recipients’ potential resistance and expedite agreement. An example is a case in the context of giving advice in teaching and supervision, where accounts can be used to highlight problems the advice is needed for (problem-account) or the benefits of following the advice (benefit-account) (Waring, 2007).

In addition, accounts can be used as a resource for conveying the speaker’s knowledge (Waring, 2007). In clinical decision-making, professionals typically appeal to their expert knowledge, whereas patients emphasize their lifeworld knowledge and personal experiences. For example, doctors use accounts when giving treatment recommendations (Angell & Bolden, 2015; Stivers et al., 2018), while patients account for rejecting the doctor’s recommendations (Landmark et al., 2015; Lindström & Weatherall, 2015). Our study focuses on accounts provided by staff members who are experts in terms of knowledge about dementia care and the prerequisites of restraining and who bear the responsibility for the resident’s treatment. However, the family members possess more lifeworld knowledge about the resident, and their connection with the residents is personal.

## Data, method, and ethical considerations

The data analyzed in this study is confidential and consist of 15 video- or audio-recorded care plan meetings (14 h and 8 min) from six Finnish nursing homes in 2020–2021. Care plan meetings concern the resident’s condition and care, the nursing home’s operating principles, and practical matters, such as clothing and billing. The Ethical Committee of the University of Turku approved the study (decision 47/2019), and the ethical guidelines of the Finnish National Board on Research Integrity (2019) were followed accurately.

Convenience sampling was utilized by contacting all nursing homes within a selected region with the help of the local Memory Association. Six units volunteered for data collection, four from the public and two from the private sector, and they were all accepted. Participation in the study was voluntary for all the informants. All nursing home staff members and family members received oral and written information about the study and signed a written consent form. The participation of the residents depended on the residents’ condition and willingness as well as the nursing homes’ meeting protocol, and required agreement from the residents’ family members. As the data were collected during the Covid-19 pandemic, the number of participants in care plan meetings was limited, and all participants except the residents were obliged to use face masks. Since researchers and assistants were not allowed in the room during the meeting, two cameras were placed on tripods.

Each meeting in our data concerns a different resident. There were 1–2 family members and 1–3 nursing home staff members (e.g., practical nurse, head nurse, manager) in each meeting. The family members either were guardians of interest nominated in the resident’s care will, or had agreed to act as contact persons for the residents. All the residents had a memory disorder diagnosis and severe dementia, and they were considered to be unable to represent themselves without substantial assistance. However, even assisted participation was rare: residents participated in only three of the meetings; in two of them the resident was absent or asleep during the discussion of restrictions, and in one, restrictions were not mentioned. Thus, our data only contains discussions about restrictions conducted by professional caregivers and staff members.

The data were subjected to conversation analysis (CA; see Sacks, 1992; In Sidnell & Stivers, 2014). This micro-analytic and empirical method has been successfully applied for research on interactions involving populations with cognitive and interactional deficits, such as dementia (for an overview, see Wilkinson, 2019). Of the studies focusing on dementia and interaction, a considerable number explore the context of long-term care (e.g., Jansson & Plejert, 2014; Lindholm, 2016; Rasmussen et al., 2019).

The data were transcribed using the conversation analytic transcription method (Jefferson, 1983; Hepburn & Bolden, 2012). When deemed relevant, information about embodied behavior was included in the transcripts. A list of the transcription symbols is given at the end of the paper.

The data analysis software NVivo 12 was utilized in the formation of the collection. In the initial phase of the analysis, 40 instances in which any kind of restraint was mentioned were identified in the transcripts. After a preliminary analysis, we limited our focus to sequences dealing with physical restraints, as they were discussed explicitly in connection with restricting the resident, whereas the use of chemical restraints could also be mentioned in an “en passant” manner alongside other information related to the resident’s medication. This limited the collection to 22 sequences. Physical restraints were either presented to family members in the context of discussing the resident’s status or addressed as a separate topic on the agenda. In the last phase of the analysis, we focused on the dynamics of the interaction around physical restraining. We analyzed how the staff members accounted for the use of restraints and how agreement with family members was pursued. Special attention was paid to the family members’ access to information and the possibilities to participate in the decisions. The authors discussed all analytic points throughout the process. Another member of the research project, Professor of Gerontologic Nursing Science Riitta Suhonen, was consulted with regard to legislation and statutes on nursing home residents’ self determination.

## Analysis

In discussing the use of physical restraints in care plan meetings, there is a clear association with handling something unfavorable—that is, limiting the resident’s autonomy. Simultaneously, restraining should, by definition, be a better choice than the harm it is intended to avoid. This conflict of doing something that is unfortunate but beneficial shows in the way the topic is handled: the nursing home staff members stress the minimal and legitimized use of restraints and distance themselves from concrete restraining actions. In their accounts for the use of restraints, the staff members highlight the potential threats to the residents if no limitations are used, as well as the potential benefits of restraining. The problem and benefit accounts in our data are often intertwined, as avoiding harm implicitly indicates promoting safety, and vice versa. The family members, on their part, respond to the staff members’ accounts with overt agreement, as protecting the well-being of the resident is their primary concern.

In Excerpt 1, two adult daughters of a resident (F1 & F2) are informed about the use of a safety belt. Before the excerpt, the group discussed the resident’s restlessness, which was one of the main reasons for their transition to long-term care. The resident’s personal nurse (PN) then announced that the next title on the agenda was “Restrictions.” The head nurse (HN) starts the discussion by addressing the possible epistemic asymmetry concerning the terms used in the resident’s documents: HN explains what having a *safety belt permission* means in practice.

Excerpt 1a. (F = family member, M = nursing home manager, HN = head nurse, PN = personal nurse)


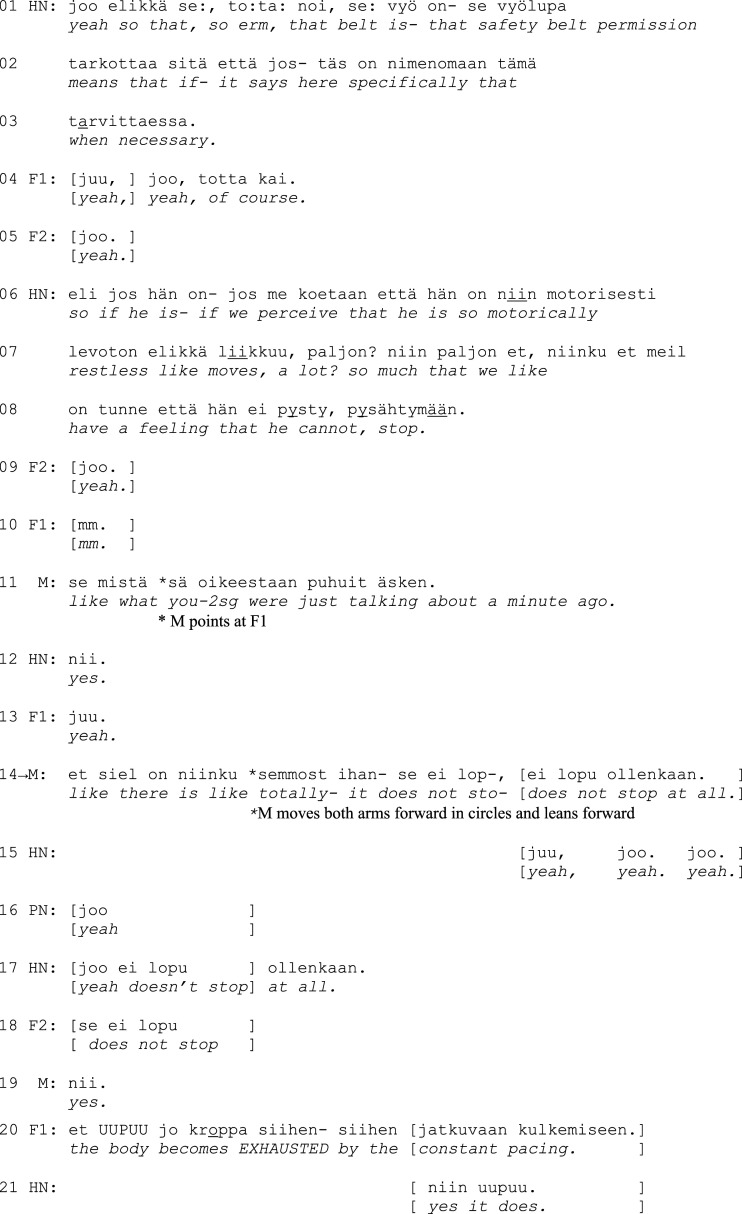


First, the head nurse quotes the wording *when necessary* in the document, highlighting that the use of the safety belt is situational and considered. The turn contains hesitation markers like restarts, elongated pronunciation (*se:*, *to:ta:*), and particles (*elikkä* ‘so, ergo’, *tota noi* ‘so, erm’) that are common when talking about sensitive matters that need to be approached with caution (*perturbations*, Linell & Bredmar, 1996). The family members express understanding and overt agreement with the introduction (*yeah, of course, yeah*, lines 4–5).

Then, the head nurse provides a problem-account: HN depicts a situation in which the resident behaves restlessly and is seemingly unable to stop pacing around. Again, the head nurse corrects their own choice of words, replacing a categorical expression *if he is* with *if we perceive that he is*, which emphasizes the staff members’ professional task of monitoring the resident. The nursing home manager then ties the head nurse’s description to the family members’ earlier accounts of the events before the transition to the nursing home (l. 11), creating a clear connection between the professional observations and the family members’ own experiences. The manager also upgrades the description of restlessness to something that *does not stop at all*, which underscores the resident’s inability to control their own actions. In addition, the manager enhances the recognizability of the situations with an embodied depiction of the resident’s behavior (l. 14).

The family members respond by conveying their recognition of the problematic situation and affiliating with the staff: they produce agreement tokens and a partial repetition (*does not stop*, l. 18) and even complete the problem-account by verbalizing in a loud voice the result of not being able to stop: *the body becomes EXHAUSTED* (l. 20). After an agreement on the problem has been reached, the head nurse continues by describing the restraint in practice (1b):

Excerpt 1b.


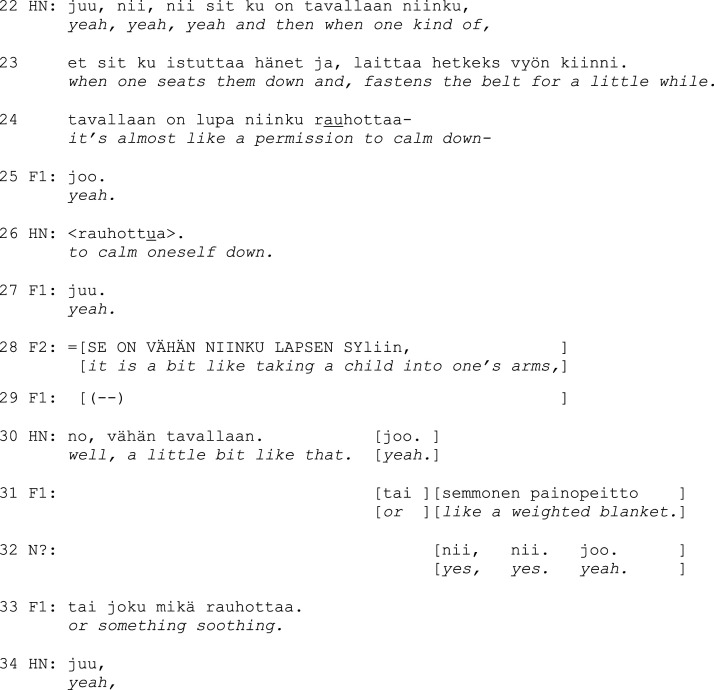


The head nurse talks about fastening the resident to a chair, using a zero-person construction, which involves a third-person verb form that has no overt subject (l. 22–24, on zero person in Finnish, see Laitinen, 2006). This leaves the agent of restraining implicit and distances the head nurse from the action. The focus is then turned to the resident’s experience, which the head nurse describes as having *permission to calm oneself down* (l. 24, 26). The family members seem to interpret this as a benefit-account, since they upgrade the usefulness in their next turns. One compares the safety belt to an embrace (l. 28) and the other compares it to a weighted blanket (l. 31), both of which are notably positive analogies to being restrained against one’s own will. Although the head nurse downgrades these notions (*a little bit like that*, l. 30), it seems clear that all parties agree that restraining is worthwhile when the resident gets restless. After the extract, the head nurse informs the family members about other restrictions, and the manager explains that their necessity will be regularly reviewed. The family members express agreement with the institution’s protocol throughout.

Excerpt 2 is also about the use of a safety belt but from another meeting. This time, the topic becomes relevant when the staff members inform the resident’s son (F1) and husband (F2) about the resident’s current health. Before the extract, the manager says that there has been a significant decline in the resident’s balance and mobility due to the progression of the memory disorder and that the resident needs more support than before. When the manager mentions that the resident needs help with moving, the head nurse introduces the need for restriction:

Excerpt 2.


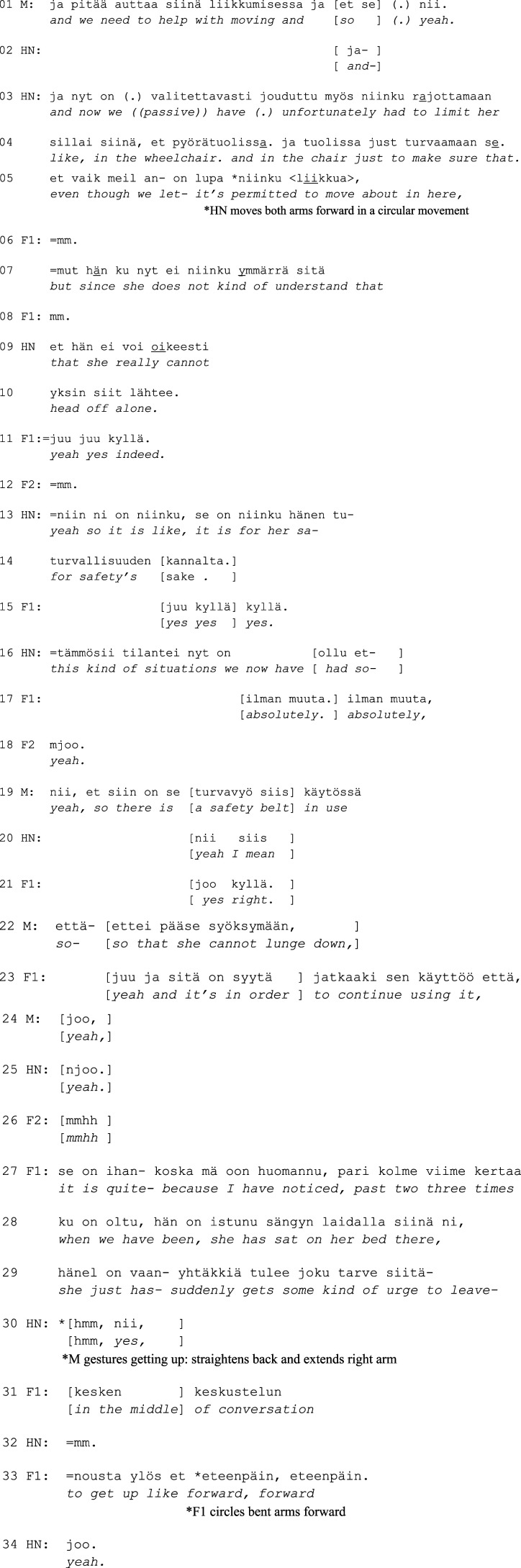


In Excerpt 2, the head nurse uses the information about the resident’s declining state as preparation for more unfortunate news, that is, the restraining (*and now we have* ***unfortunately*** *had to*, l. 3) (on breaking bad news, see Maynard, 2005). After explaining that moving around in the facility in general is accepted, HN offers a problem-account for the restriction; the resident does not understand the limits of their own physical capability (l. 5, 7, 9). HN also adds that there have already been risky situations (l. 16), which acts as evidence of the necessity for taking safety measures.

What is notable is the way family members receive this information. First, F1 responds with *mm* (l. 6, 8), which conveys listening. Bad news receipts are typically produced in this kind of restrained manner, and they may also contain elements of regret, reluctance, or disbelief (Maynard, 2005). Here, however, the staff members’ accounts seem to make the news more acceptable and less sensitive, and F1 responds to them with overt agreement. The responses contain reduplicated agreement tokens (*juu juu*; *kyllä kyllä*; *ilman muuta, ilman muuta)*, and they are produced without delay and even in overlap (l. 11, 15, 17).

The manager clarifies that the reason for the safety belt is to prevent the resident from falling (l. 19, 22). In overlap with M’s turn, F1 urges the staff to continue using the belt (l. 23), after which they shares their personal experiences of risky situations with the resident (*I have noticed*, l. 27–29). F1 describes the resident’s actions as sudden, and both F1 and the manager depict the resident’s spontaneous movements with gestures (l. 30, 33). Thus, similarly to Exc. 1, shared understanding is achieved by connecting the professionals’ perceptions with the family members’ experiences with the resident.

However, family members’ knowledge does not always match or complement the professional view, nor do family members always have experiences compatible with ongoing challenges. Our next excerpt is from a one-on-one meeting between a nurse (N) and a resident’s adult son (F). Similarly to Exc. 2, a discussion of the restrictions comes after information on the resident’s current health. Before the extract, the nurse informed the resident’s son that it had become difficult to communicate with the resident and that there had been some conflicts between the nursing staff and the resident. N then discloses the use of physical restraints:

Excerpt 3a. (only audio)


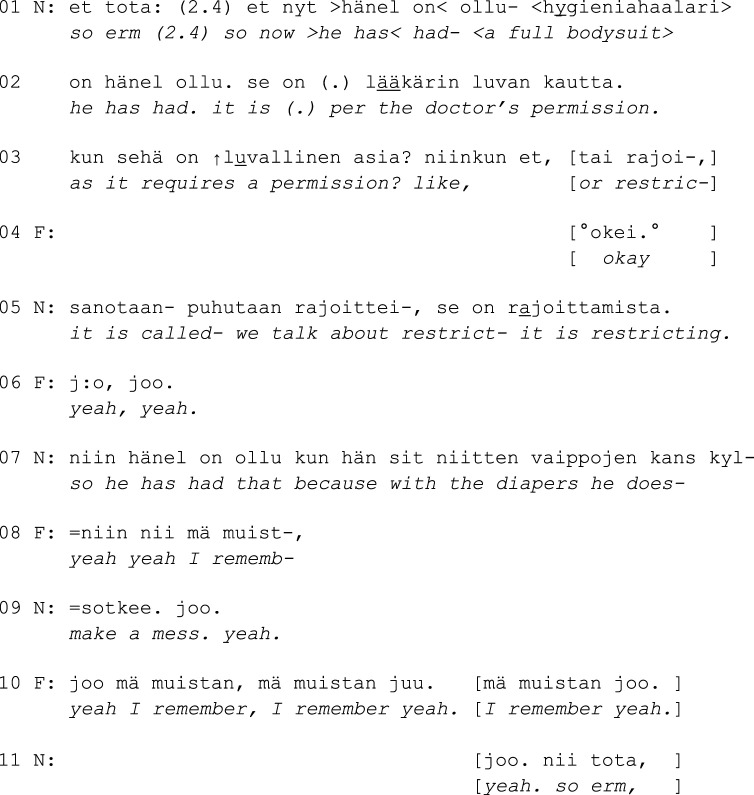


In Exc. 3a, N first tells F that the resident’s doctor has permitted the use of a full bodysuit. Again, the formatting fades the staff members’ involvement in the restraining actions: the resident is not put in a suit but *has* a suit (l. 1). Nevertheless, the nurse explicitly informs F that they are talking about restricting (l. 5), which shows that the family member is not expected to know that using a full body suit is not a mundane procedure in a nursing home environment and that this information is important to understand.

The nurse then starts a problem-account (*because with the diapers he does-*, l. 7), which is interrupted by F’s response *yeah I rememb(er)*. In effect, when the nurse nevertheless completes the account (l. 9, *make a mess*), F repeats the same response three more times (l. 10). By highlighting his *remembering* F signals being made aware of the situation before rather than having a personal experience about it. Repeating an utterance word-for-word can also indicate that the co-participant has not taken this utterance into consideration to an adequate degree (Rauniomaa, 2008). In this case, the family member’s repeated notion of remembering may convey that the family member is reluctant to hear more about the topic. The nurse then moves on to inform F about the use of a safety vest, which in this case refers to a flexible vest attached to the resident’s bed. The transition to the topic conveys hesitation (l. 11–12), which seems to signal difficulty disclosing more sensitive information to family members.

Excerpt 3b. (only audio)


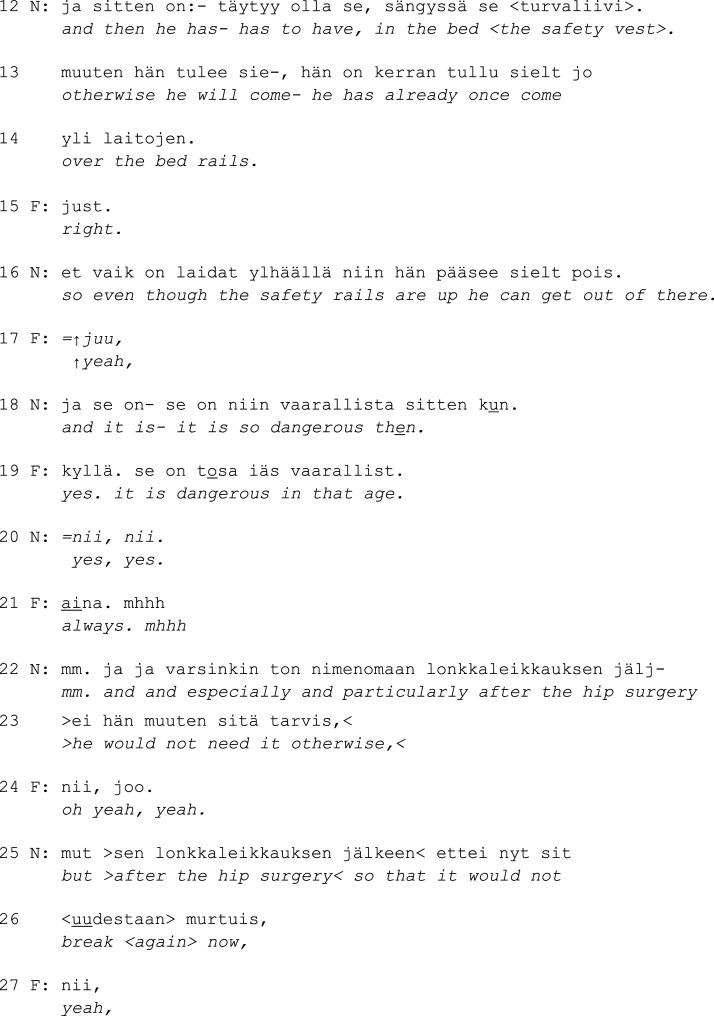


Again, the nurse depicts the restriction as something that the resident has to *have* rather than something that is imposed on them (l. 12). N accounts for the situation with a problem: the resident getting out of bed. This is received as new information by F, as *just* ‘right’ is a change-of-state token in Finnish (Koivisto, 2017; Heritage, 1984). When the nurse explains getting out of bed as being dangerous, F agrees. This time, however, the family member’s understanding of the danger differs clearly from the nurse’s: F connects the need to stay in bed to the resident’s old age (l. 19) and generalizes the risk to other people as well by adding *always* in an accented tone (line 21). The nurse responds to F with *mm*, which conveys only weak agreement (see Sorjonen, 2001) and then explicitly links the danger to the resident’s recent hip surgery and emphasizes that otherwise there would be no need for the safety vest (l. 22–23, 25–26). Hence, the nurse does not settle for F’s agreement but ensures that they understand the institutionally valid prerequisites of restricting before moving on with the meeting’s agenda.

To conclude, the nursing home staff orients to the information about physical restraining as sensitive and unfortunate but also as something that the family members must hear and become aware of. However, the staff members shift the focus from the restraining actions to the problematic situations leading to restraining and seek family members’ agreement on the reasoning. We will contemplate what this kind of interactional dynamics means in terms of the resident’s care and rights in the Conclusions section.

## Discussion: Towards a more sustainable practice

This study provides a concrete perspective on phenomena that are relevant with regard to dementia care, self-determination, decision-making, and family member involvement. As our qualitative study is based on a rather small sample collected from six Finnish nursing homes, it is important to note that care plan meetings can be arranged in varied ways and that some nursing homes do not arrange such meetings at all. In fact, the meetings in the data can also be regarded as somewhat exceptional as they were recorded during the first years of the Covid-19 pandemic when visits to nursing homes were limited in different ways. For example, there were different rules regarding the number of visitors and the duration of visits, and meetings were often postponed. Second, the safety measures used in the meetings (number of participants, distance between participants, use of face masks, possibilities of offering refreshments) were new at the time. Hence, the exceptional times may have affected both the interaction and the emotional experience of the participants (see, e.g., Paananen et al., 2021). Another limitation of our study is that we did not have the opportunity to study decision-making involving persons with dementia, as the residents were excluded from the care plan meetings.The data is nevertheless authentic in that it consists of naturally occurring interactions that would have taken place regardless of the study, and the procedure of recording interaction has not been shown to decline in quality (Parry et al., 2016).

A previous study by Saarnio (2008) found that Finnish family members’ conceptions of the necessity of restraining nursing home residents were overscaled. The study also found that family members had too much influence on the restriction protocol. While the authentic discussions in our study confirmed the family members’ tendency to support restraining as an effective method to guarantee the resident’s safety, they also illustrated that family members were at the periphery of decision-making (cf. Weiste et al., 2020): the decisions on restraining were presented to the family members as ready-made decisions. However, as our data consist only of recorded care plan meetings, we cannot be entirely certain that the family members had not been consulted via telephone or mail prior to the meetings. With respect to the current regulations and ethical guidelines, it would be advisable to involve the family members of residents with late-stage dementia in the decision-making about restraining as early as possible, or to adjust the protocol in the care plan meetings with the family members (see also Puurveen et al., 2018).

As limiting a person’s mobility can have serious negative consequences (Gastmans & Milisen, 2006; De Bellis et al., 2013) and there is a risk that restraining may become a daily practice instead of an occasional solution in geriatric care (see Möhler & Meyer, 2014), alongside highlighting the minimal use of physical restraints, staff members should involve family members—as well as the residents—in designing *how* to minimize the use. For example, it would be useful to discuss what kinds of things the resident finds upsetting and comforting, and which restraining options both the residents and their family members consider least invasive. In general, more attention could be paid to the residents’ experiences and the family members’ lifeworld knowledge about the resident. We believe that including residents in the meeting would support patient-centered care, even if their interactional contributions are limited due to dementia (cf. Daly et al., 2018; Kitwood, 1997). First, inclusion would enable the resident’s participation during moments of lucidity, and second, having the resident physically present in the situation could highlight the fact that the decisions concern a living, feeling human being.

Finally, it is important to acknowledge that the vulnerability of persons with dementia is situational and contingent on environmental and institutional structures and policies (cf. Bracken-Roche et al., 2017). Research can offer valuable insights for developing more sustainable and ethical policies and protocols, but concrete changes demand interdisciplinary collaboration in policymaking and education and a long-term commitment.

## Conclusion

Analysis of the data shows that reaching agreement with residents’ family members on the use of physical restraints is achieved relatively easily. First, staff members inform the family members about the principles of restraining (i.e., having a doctor’s permission, careful monitoring), and then, they account for the use of restraints by highlighting the problems that can be avoided and the benefits that can be achieved by limiting the resident’s actions. Staff members’ accounts highlight the aim to protect the resident’s health and safety, which is expectedly in the interest of the family members as well. In effect, family members respond to the accounts immediately, even in overlap, with overt agreement.

In addition to fostering agreement, the practice of informing and accounting shifts the focus away from the concrete actions of restraining to the causes that lead to restraining. In the data, staff members position themselves as regulators who monitor the situation, while the execution of restraining is portrayed in general terms (*one fastens the belt*) or agentlessly (*he has had a full bodysuit*). Shared understanding is primarily established about problematic situations: the staff and family members connect their observations (Exc. 1a) and share short anecdotes about events and incidents with the resident (Exc. 2). In this endeavor, both parties are treated as *knowing* in relation to the problems arising from the resident’s memory disorder symptoms, and the two domains of knowledge are combined (cf. Heritage, 2012). If a family member’s understanding is not compatible with the professionals’ (Exc. 3), the staff members can level the gap in knowledge by offering more information.

While the shared understanding of the causes leading to restraining is secured by the staff members in the discussions, family members’ understanding of the prerequisites of restricting is not as carefully addressed. Whereas staff members stress the need for official permission, careful consideration, and minimal use of restraints, family members tend to produce “upgraded” responses that make the restrictions more generally acceptable (*it is a bit like taking a child into one’s arms; it’s in order to continue using it; it is nothing, even if it is used all the time*). Although family members’ upgraded views convey strong agreement and trust in the nursing home staff, which from the viewpoint of the interactional relationship are positive, they also contradict the regulations: restraining should always be a situational and temporary solution (Valvira, 2021). Promoting the use of restraints raises a concern that family members may perceive restraining solely as recommendable protection and that staff members may be prone to accept this kind of view. It is only in Exc. 3 that the staff correct the family member’s upgraded view after initial acceptance.

It should also be noted that family members’ strong agreement with the restriction protocol could convey a desire to maintain friendly connections with the staff. A study by Hoek et al. (2021) of family members’ attitudes toward interactions with healthcare practitioners showed that family members strive to remain on “the same side” with the staff even when they have something to say. Family members may refrain from asking questions and making requests to avoid being labeled “moaners.” It should also be noted that expert opinions could be highly appreciated by family members, and family members were probably grateful in general for acquiring a place in a nursing home for their next of kin in the current societal situation. The threshold for family members to disagree with the staff, let alone suggest alternative solutions, on matters that require knowledge about legislation and professional guidelines is thus presumably high.

To conclude, the major issues with the professional practice of informing and accounting from the viewpoint of the residents’ agency are as follows. 1) The family members’ role is to accept a decision that has already been approved by the authorities. 2) Agreement is pursued about the goals of restraining, whereas the methods are not negotiated. In this kind of operational model, the residents’ own experiences and intentions receive little attention, and family members’ opportunities to advocate for the residents are limited (cf. Finnish Act on the Status and Rights of Patients, § 6). In fact, discussion about alternative solutions or softer methods is missing in our data; family members are not asked what kinds of things could potentially prevent agitation or restlessness or what could be done to calm the resident down in problematic situations.

## Appendix

### Transcription symbols

[ ]: overlapping talk
=: latching
(.): micro pause
(0.1): timed pause
-: cut-off of preceding sound
extension of a sound
°word°: quieter voice
↑: rise in pitch
?: rising intonation
,: continuing intonation
.: falling intonation
(--): transcriber could not hear what was said
*waves: action or gesture
